# Cross-Cultural Register Differences in Infant-Directed Speech: An Initial Study

**DOI:** 10.1371/journal.pone.0151518

**Published:** 2016-03-16

**Authors:** Lama K. Farran, Chia-Cheng Lee, Hyunjoo Yoo, D. Kimbrough Oller

**Affiliations:** 1 Communication Sciences & Disorders, University of West Georgia, Carrolton, Georgia, United States of America; 2 Communication Sciences & Disorders, University of Memphis, Memphis, Tennessee, United States of America; 3 Konrad Lorenz Institute for Evolution and Cognition Research, Klosterneuburg, Austria; University of Portsmouth, UNITED KINGDOM

## Abstract

Infant-directed speech (IDS) provides an environment that appears to play a significant role in the origins of language in the human infant. Differences have been reported in the use of IDS across cultures, suggesting different styles of infant language-learning. Importantly, both cross-cultural and intra-cultural research suggest there may be a positive relationship between the use of IDS and rates of language development, underscoring the need to investigate cultural differences more deeply. The majority of studies, however, have conceptualized IDS monolithically, granting little attention to a potentially key distinction in how IDS manifests across cultures during the first two years. This study examines and quantifies for the first time differences within IDS in the use of baby register (IDS/BR), an acoustically identifiable type of IDS that includes features such as high pitch, long duration, and smooth intonation (the register that is usually assumed to occur in IDS), and adult register (IDS/AR), the type of IDS that does not include such features and thus sounds as if it could have been addressed to an adult. We studied IDS across 19 American and 19 Lebanese mother-infant dyads, with particular focus on the differential use of registers within IDS as mothers interacted with their infants ages 0–24 months. Our results showed considerable usage of IDS/AR (>30% of utterances) and a tendency for Lebanese mothers to use more IDS than American mothers. Implications for future research on IDS and its role in elucidating how language evolves across cultures are explored.

## Introduction

### Background

Language is both a biological and a social phenomenon [1] and a (perhaps *the*) hallmark of what makes us human. While the language capacity itself may be inherited, languages—including how meanings emerge as well as how they are shared and understood—are passed on through cultures.

One central, persistent question in the quest for language origins is how infants across cultures of the world develop such diverse languages. Modern biology makes clear that the evolution of any living system depends on the orchestration of various subsystems interacting at different levels and timescales, and relying heavily on environmental input [2]. In the case of language, a special kind of environmental input is presented to very young infants through interactions with their caregivers. These interactions reflect cultural “niche construction”–[3,4], yielding a human-specific environment for language learning, created by humans in each culture and passed on through generations.

However, theories and proposals aimed at elucidating the origin of language have paid scant attention to the role of human culture [1,5]. Recently, [6] proposed the linguistic niche hypothesis, according to which language structure is shaped by the culture in which language is learned. Their proposal focuses both on adult second-language learning and child first-language learning and on the extent to which cultures may differ in how language evolves in vocal interaction. Our focus here is on the possibility of culturally-specific input differences for language-learning in infants.

Accordingly, we sought to study the role of environmental input in two different cultural groups in an effort to describe amounts of infant-directed speech (IDS) produced by mothers as they interacted with their infants. Across many languages and cultures, IDS has been described as a special way of addressing infants, characterized by salient acoustic features such as exaggerated prosody [7–10], longer vowels [11], and shorter and less complex utterances [12]. This sort of speech has sometimes been termed “motherese”[13,14] or “baby talk” [15]. Still, the term IDS has not always been used specifically to invoke acoustic features that differentiate it from adult-directed speech (ADS). We shall employ the terms “baby register” (hereafter IDS/BR) to refer to speech including such differentiating acoustic features and “adult register” (IDS/AR) to refer to speech that does not include those features, even in cases where the speech in question is indeed IDS. The literature on IDS, though not having quantified use of different registers, seems to suggest that especially at young infant ages, IDS/BR is much more frequent than IDS/AR.

The potential importance of the distinction is clear: parents across cultures do not speak with their children using the same vocal range or register throughout the day. They sometimes switch between registers rather seamlessly, one moment addressing their infant in IDS/BR (as in the case of looking at the infant and telling her how cute she is) and another moment using IDS/AR (as in the case of telling the infant it is time for a bath).

IDS/BR is thought to have evolved as a species-specific adaptation [16, 17], where parents intuitively attempt to adjust their speech to infants’ developmental stage and to respond to infants’ initiations of communication bids in a particularly engaging and affectively-charged manner. Empirical evidence supports the idea that IDS/BR plays a role in attracting and maintaining infant attention during face-to-face interaction [18, 19] (and in fostering important gains in socio-cognitive and language development [20, 21].

A great deal of cross-cultural research on IDS exists, and many studies have been concerned with effects IDS may have on infants’ development across domains including receptive and expressive language [11, 22–29], cognition [30], and vocalizations or speech [31]. Other work has focused on quantifying the acoustic and affective content of IDS compared to that of ADS [32, 33, 34] without drawing a specific distinction between usage of IDS/BR and IDS/AR *within* IDS. This lack of quantification of register usage results in the presumably unintentional implication of IDS as a monolithic, static construct (but see, [35]). Still, research has pointed out that IDS diminishes in frequency of occurrence as infants get older [36], and this implies, not that parents talk less to their older infants, but that *IDS/BR* is used less with older infants [37, 38].

Recent evidence suggests that IDS is not used uniformly across cultures [39], nor is it unitary in form. Instead, IDS appears to be a multidimensional, dynamic entity with numerous functions that change in response to contextual demands (such as providing information versus sharing affect), differences in the acoustic properties of languages (tonal versus non-tonal), infant’s developmental level, age, and presence of or risk for disorders. Poignant support for a culturally-specific conceptualization of IDS comes from reports contesting the notion of universality of IDS on the grounds that, in certain cultures, adults appear not to use a special register at all when addressing young infants [40, 41]. This research involves little to no quantification, so it is clear that more empirical work is called for.

### Purpose

In the present study, we focus on cross-cultural similarities and differences in the use of IDS among a group of Lebanese Arabic-speaking and a group of American English-speaking mothers as they communicated with their 0–24 month infants, and we seek answers to the following research questions:

1. What is the relative frequency of usage of IDS/BR and IDS/AR in parents of these Lebanese Arabic-learning infants and parents of these American English-learning infants?
2. Do parents of these Lebanese Arabic-learning infants and parents of these American English-learning infants differ in IDS Utterances per Minute (or alternatively IDS Seconds per Minute) spoken to their infants?
3. Do parents of these Lebanese Arabic-speaking and American English-speaking parents differ in the relative use of IDS/BR and IDS/AR within their respective cultures as well as across cultures?
4. What is the impact of language, register type, and infant age on IDS Utterances per Minute (or alternatively IDS Seconds per Minute)?

Answers to these questions should better characterize the cross-cultural nature and variability of IDS and help to elucidate how language evolves across cultures. The uniqueness of our approach, to our knowledge, lies not only in the comparison of these particular cultural groups, but also in the fact that we quantify the utilization of IDS/BR and IDS/AR across the languages.

## Methods

### Participants

This research was approved by the Institutional Review Boards at the University of West Georgia and the University of Memphis. All parents completed an informed written consent to participate in the study. The study combines two independently developed recording sets, one for Lebanese-Arabic families and one for American-English families.

Arabic and English served as target languages because they are both among the most widely spoken languages in the world, with English being second and Arabic fourth [42]. In addition, English and Arabic come from very distinct language families—Indo-European and Semitic, respectively—with dramatically different morpho-syntactic structures and phonologies. One distinguishing characteristic of Arabic is diglossia [43], a sociolinguistic phenomenon which consists of two forms used side-by-side for different functions: Informal/Ammiya, used for various social and communicative purposes, and formal/Fusha used for formal purposes, including reading, writing, and formal discourse [42]. Since motherese is essentially social and informal, Lebanese mothers use Ammiya just as American mothers use conversational English when interacting with their infants. In both cases, the IDS/BR is characterized by wide prosodic and affect variations.

The first author had recorded parent-infant interactions in Lebanon, where virtually everyone’s first language is Arabic. The Arabic-speaking Lebanese mothers and their infants (12 male and 7 female; age range 0–24 months) were recruited from two private and two public pediatric clinics in Lebanon. All mothers spoke to their infants in Ammiya.

In collaboration with the Infant Vocalizations Project at the University of Memphis, where an archive of roughly similar recordings exists based on prior research, we sought to develop a maximally matching sample from English-speaking families in the United States to parallel the Lebanese sample in participant demographics, namely infant age, infant gender, and maternal education. The resulting study participants consisted of 38 mother-infant dyads (19 Lebanese who spoke Arabic as their first language in addition to French or English as their second language; and 19 American who spoke English as their first and only language) Table 1. The American mothers and their infants (9 male and 10 female; age range 1–24 months) had been recruited originally in two University of Memphis longitudinal studies on infant vocal development. Infants from both cultures were typically-developing with no reported complications. Maternal education for both the American and the Lebanese samples ranged from high school to graduate school Table 2. Overall, the two cultural groups did not differ significantly in the distribution of maternal education, age, or gender, a point we will address in the results section.

**Table 1:** Demographic Characteristics of Participating Infants.

Age	Gender	Gender
in months	Arabic-learning infants	English-learning infants
0	M	
1		F
5	F	F
6	M	F
6	F	F
6	M	M
8	F	F
9	F	F
10	M	F
10	M	M
11	M	M
12	F	F
12	M	F
12		M
13	M	
16	M	M
16		M
17	M	
18	M	F
20		M
21	F	
21	F	M
24	M	M

**Table 2:** Descriptive Statistics.

	American	Lebanese	Total
	N = 19	N = 19	N = 38
Maternal Education			
Graduate	6	5	11
Undergraduate	8	5	13
High school	5	9	14
Mean Rate in Utterances per Minute			
IDS/BR (SD)	9.14 (4.57)	11.29 (4.26)	10.21 (4.5)
IDS/AR (SD)	4.21 (3.25)	6.03 (3.83)	5.12 (3.62)
Mean Duration in Seconds per Minute			
IDS/BR (SD)	10.41 (5.82)	11.19 (3.87)	10.80 (4.89)
IDS/AR (SD)	3.66 (2.61)	5.12 (3.42)	4.39 (3.09)
Mean Duration in Seconds per Utterance			
IDS/BR (SD)	1.12 (.36)	1.02 (.20)	1.07 (.29)
IDS/AR (SD)	.91 (.24)	.80 (.16)	.86 (.21)

We emphasize that the samples for this cross-university collaboration were not perfectly matched either in demographics or in recording procedures because the study was opportunistically designed after all the recordings had been obtained from both cultures.

### Procedure

The Lebanese Arabic samples were audio-recorded in the infants’ homes using high fidelity equipment (sampling rate in all cases 48 kHz) with built-in stereo microphones. Prior to the recording sessions, the Lebanese mothers were instructed by the experimenter to interact for 10 minutes with their infants as they normally did at home. Mother-infant dyads participated in the recording sessions with no children present, and only occasional interaction between the mother and the experimenter (who usually stayed in another room) occurred as necessary e.g., to help manage recording equipment. This occurred in 9 out of the 19 Arabic recordings, with minimal adult-to-adult talk (in most cases ranging between 1 and 3 utterances, with only one case with 4 utterances) per recording.

The American English samples were digitally recorded for audio (sampling rate in all cases at least 20 kHz) in TF32 [44] using wireless microphones worn on both infant and mother in the infant vocalizations laboratory at the University of Memphis. These were segments where American mothers were asked to interact with their infants as they would at home for 20 minutes and where no other persons were present except (as in the case of the Lebanese recordings) occasionally during brief intervals when an experimenter might enter briefly e.g., to adjust microphones. To maximize comparability with the Lebanese samples, we selected 10-minute segments with only parent and infant present whenever possible. Usually the first 10-minutes met the requirement and was selected. If there was any vocal interaction between the parent and the occasionally present experimenter during the first 10 minutes, the next consecutive 10-minutes was selected where there was no adult-to-adult talk. There were no consecutive 10-minute periods without any adult-to-adult talk in two of the 19 American recordings, and in those two cases, we selected the consecutive 10-minutes with the least adult-to-adult talk. These recordings included 1 and 4 adult-to-adult utterances, respectively.

### Coding

The first author, who is a speaker of Arabic as L1, English as L2, and French as L3, served as the primary coder. The second author, who is a speaker of Mandarin as L1, Southern Min as L2, and English as L3, served as the reliability coder. Both the primary and secondary coders coded all 38 sessions completely independently. The primary analysis was based on the first coder’s work, since she knew both target languages.

Mothers’ utterances were coded in PRAAT [45], which is an acoustic analysis system available as on-line freeware that allows coders to view waveform and spectrographic displays in real-time, place cursors on the screen to indicate onset and offset of vocalizations, and to determine the locations in time and durations of each parent utterance. Coders identified the onset and offset of each utterance using a breath-group criterion (i.e., one utterance per breath group, as recommended by [46]). Two rounds of training in cursor placement were conducted under the supervision of the last author (a phonetician who has provided university level training in coding and speech analysis for many years) with comparison of results from each coder in a group meeting and discussion following each round of training. The recorded materials used in this training were drawn from other recordings in the University of Memphis archives, so that the real data collection would be uncontaminated by awareness on the part of either coder of the opinions of either the trainer or the other coder regarding the samples actually used in the analysis. The two coders reached better than .8 correlation across coded training samples before proceeding to data collection.

A coding scheme was developed for this study classifying parent utterances into a number of mutually exclusive acoustically-based categories that were later collapsed for analysis into IDS/BR and IDS/AR. Utterances were treated at analysis as IDS/BR if they were judged intuitively (no acoustic analysis necessary) to include *any one* of the following nine qualities: (1) pitch or pitch range notably exceeding that of typical adult-to-adult speech; (2) long duration per syllable compared to adult-to-adult speech; (3) smooth intonation with a soothing tone, the kind of intonation described by [47, 48]; (4) sing-song pattern of rise and fall in intonation; (5) parent production of infant vocalizations such as squeals, growls, or raspberries (“protophones”, see [49]; (6) very long final syllables, even longer than the lengthened final syllables used to mark boundaries in adult speech [50]; (7) immediate caregiver imitation of infant sounds; (8) nonvocal sounds, for example (a) any isolated prominent ingressive breath, a pattern that has been observed in the Memphis laboratory as commonly used by caregivers in an attempt to elicit an infant affective response and (b) voiceless shushing; and (9) parent laughing toward the infant during talk. Although we initially included singing to the infant in our coding of IDS, we ultimately excluded it from the analyses because it occurred infrequently, and we saw no strong basis for categorizing it differentially as IDS/BR or IDS/AR. During the coding, features (1)-(6) were all categorized as a single “general” BR type, whereas (7)-(9) were given individual codes (non-vocal, immediate imitation, and laughing toward infant). Table 3 shows that the general IDS/BR type accounted for the vast majority of IDS for both mother groups. IDS/AR was coded by exclusion when utterances involved none of the 9 features.

**Table 3:** Proportion of IDS/BR Subcategories in Maternal Utterances.

	Lebanese Mothers	American Mothers
IDS/BR general	0.94	0.92
Immediate imitation	0.02	0.01
Nonvocal	0.03	0.04
Laugh with infant	0.02	0.03

This coding system is not based on the semantic content of the caregiver utterances. Instead it focuses on IDS features judged on acoustic/prosodic (i.e., suprasegmental) grounds that are thought to be shared across languages as features marking motherese. We chose them precisely to guide judgments of coders who may or may not be familiar with the target languages, and the coder agreement data to be presented below confirms that the two selected coders, with very different language backgrounds, produced quite concordant codes for IDS/BR and IDS/AR. Some of the features we used to designate IDS/BR have not been considered, to our knowledge, as characteristics of IDS in prior literature. In part the decision to include all these features as properties of IDS/BR was intended to ensure that we did not overestimate the amount of *IDS/AR*, which to our knowledge has not been directly quantified in previous cross-cultural research.

### Design and Data Analysis

The primary dependent variable in this study was the number of maternal utterances in each of the 10-minute samples expressed in IDS Utterances per Minute. We also determined the duration of maternal utterances and derived IDS Seconds per Minute for an additional analysis which sheds important additional light on the findings. The statistical design entailed a mixed multivariate within-between factorial analysis of covariance (MANCOVA) in SPSS.

### Intercoder Reliability

Session-level intercoder reliability (N = 19) was measured using Intraclass Correlation (ICC), with a Two-way Random model to compute absolute agreement [51]. For Arabic, ICC was Optimal-Excellent (IDS/BR utterance per minute = .90; IDS/AR uterance per minute = .89; IDS/BR seconds per minute = .91; IDS/AR seconds per minute = .91); and for English ICC was Excellent (IDS/BR utterance per minute = .93; IDS/AR uterance per minute = .93; IDS/BR seconds per minute = .96; IDS/AR seconds per minute = .92).

## Results

Descriptive statistics are presented in Table 2. The results for Mean Rate in Utterances per Minute show, perhaps surprisingly, a substantial amount of IDS/AR in the sample, accounting for 33% of IDS utterances (35% for Lebanese mothers and 32% for American mothers). Not a single mother failed to produce at least some IDS/AR utterances in her sample, and even for infants < 7 months of age, IDS/AR accounted for >10% of maternal talk. Similarly, the alternative analysis in terms of IDS Seconds per Minute also showed that mothers used IDS/AR quite frequently: for Lebanese mothers, an average of 31% of the time in recordings being occupied by IDS was IDS/AR, and for American mothers, 26% of the time occupied by IDS was IDS/AR.

**Fig 1:**
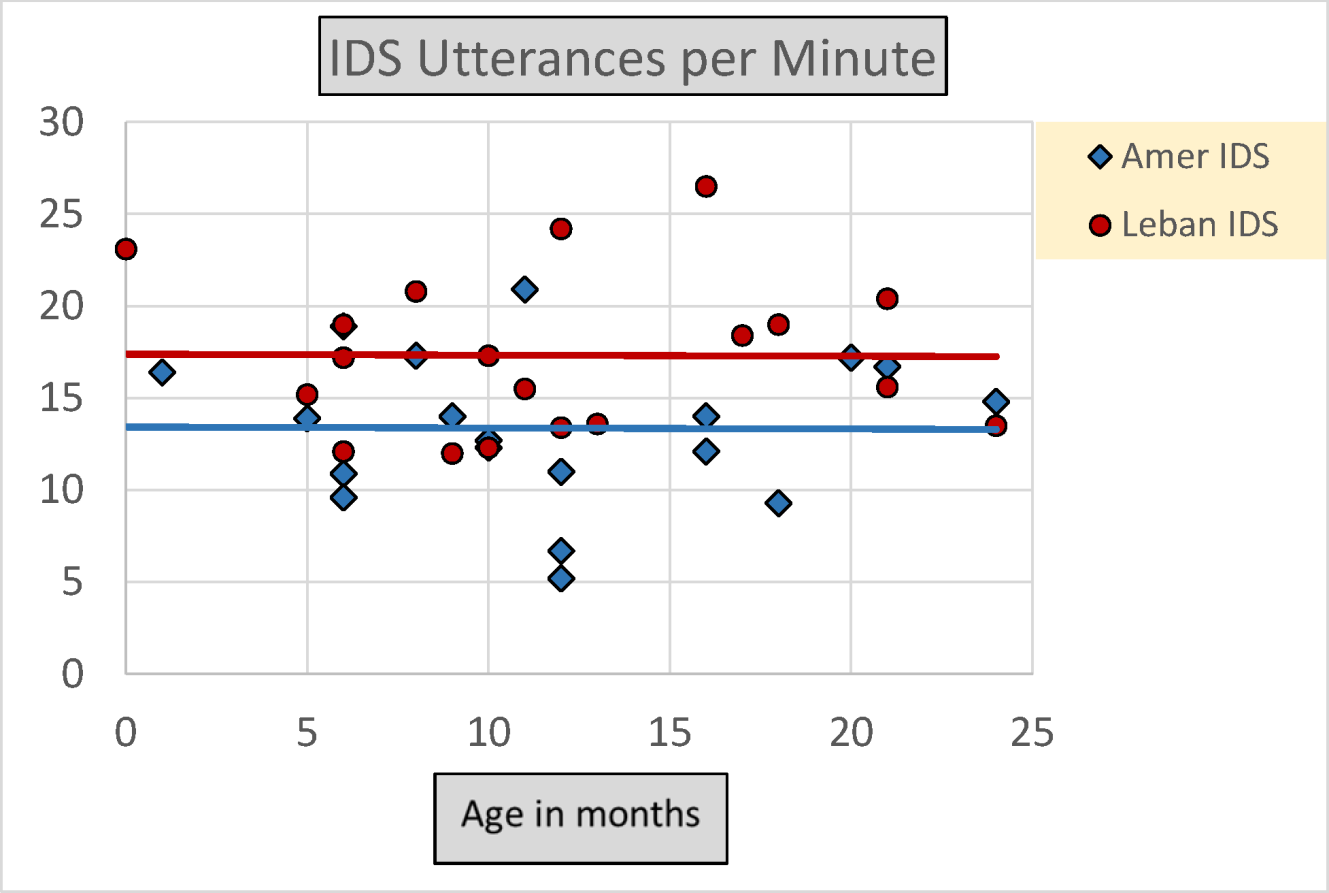
Average rate in IDS in Utterances per Minute by Language and Age. The data show that Lebanese mothers, compared to American mothers, produced more IDS Utterances per Minute when interacting with their infants in the first two years of life. The differences did not significantly vary by Age of infants (r ~ 0 for both Language groups across Age).

**Fig 2:**
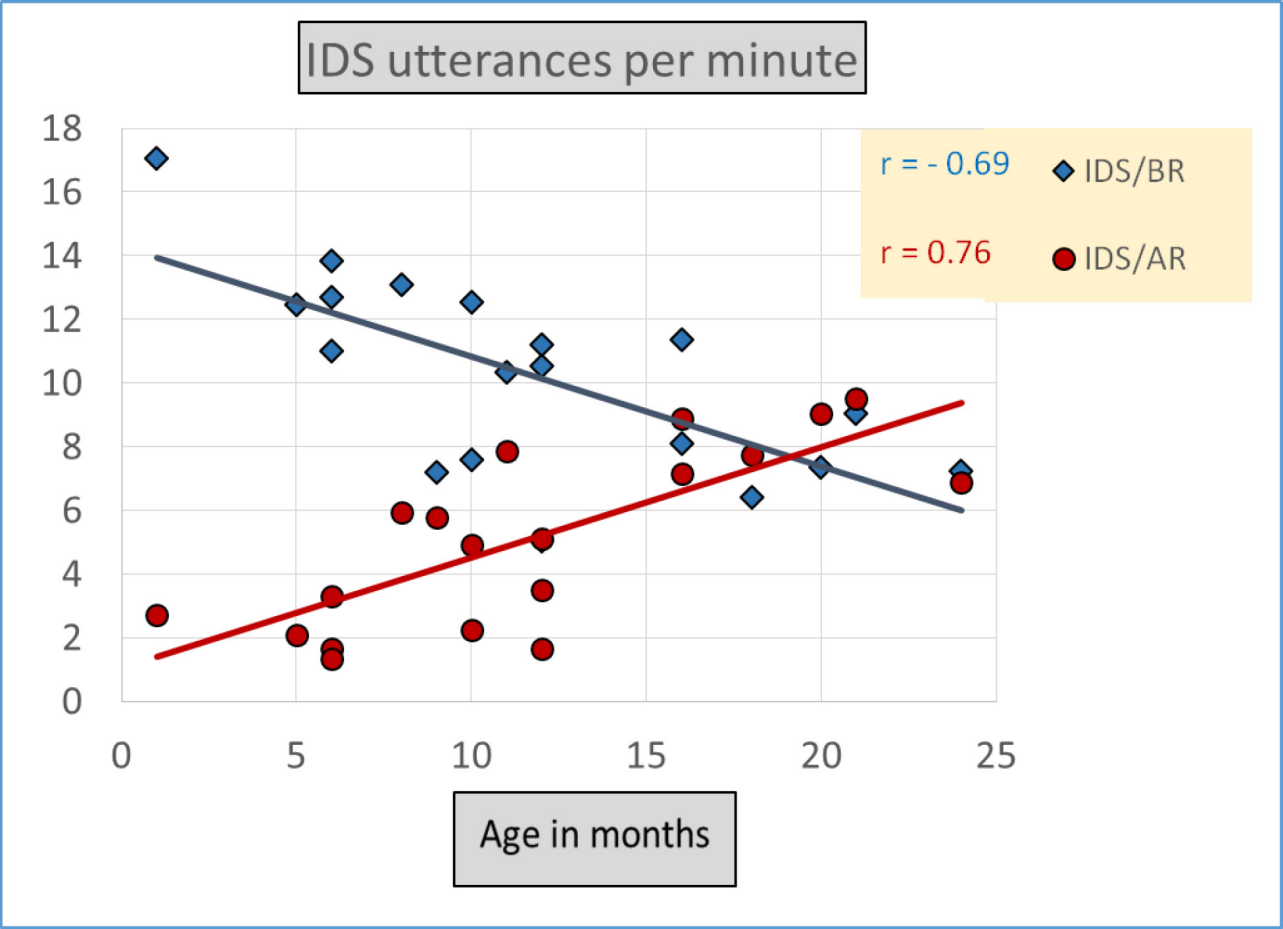
Average rate in Utterances per Minute of maternal IDS/BR and IDS/AR by Age. Both groups showed more *IDS/BR* in Utterances per Minute than *IDS/AR*. The data also show that IDS/BR was *higher* at younger than older ages and vice versa for IDS/AR.

The tabulated results for Rate in Utterances per Minute also indicate that Lebanese mothers, compared to American mothers, produced more IDS, a fact reflected in Fig 1 as well. A mixed multivariate between-within analysis of covariance (MANCOVA) was conducted for the data on Rate in Utterances per Minute to explore the impact of Language as the between-subjects factor and Register type as the within-subjects factor on the number of maternal Utterances per Minute, using Age as a covariate. There was a statistically significant effect of Register, Wilks’ Lambda = .44, *F* (1, 34) = 45.55, *p <* .001, partial eta squared = .57, reflecting more IDS/BR than IDS/AR in both Language groups and a statistically significant interaction of Register by Age, Wilks’ Lambda = .62, *F* (2, 33) = 21.54, *p <* .001, partial eta squared = .38, reflecting the fact that mothers in both Language groups produced relatively more IDS/BR Utterances per Minute to their younger infants, while they produced relatively more IDS/AR to their older infants (Fig 2). No significant interaction of Register by Language was found, Wilks’ Lambda = .99, *F* (3, 32) = .05, *p* = .82, partial eta squared = .00. There was a statistically significant effect of Language, *F* (3, 32) = 8.43, *p* = .006, partial eta squared = .19, with the Lebanese mothers using more IDS Utterances per Minute across both Registers than the American mothers (Figs 1 and 2).

We conducted covariance analyses to determine if the observed differences within and across Languages could have been driven by the somewhat uneven distribution of maternal Education and Gender in the American and Lebanese samples. Results revealed no statistically significant effect of maternal Education or Gender on the number of maternal Utterances per Minute. All effects from the main analysis remained significant when Maternal Education and Gender were entered as covariates in these additional analyses.

**Fig 3:**
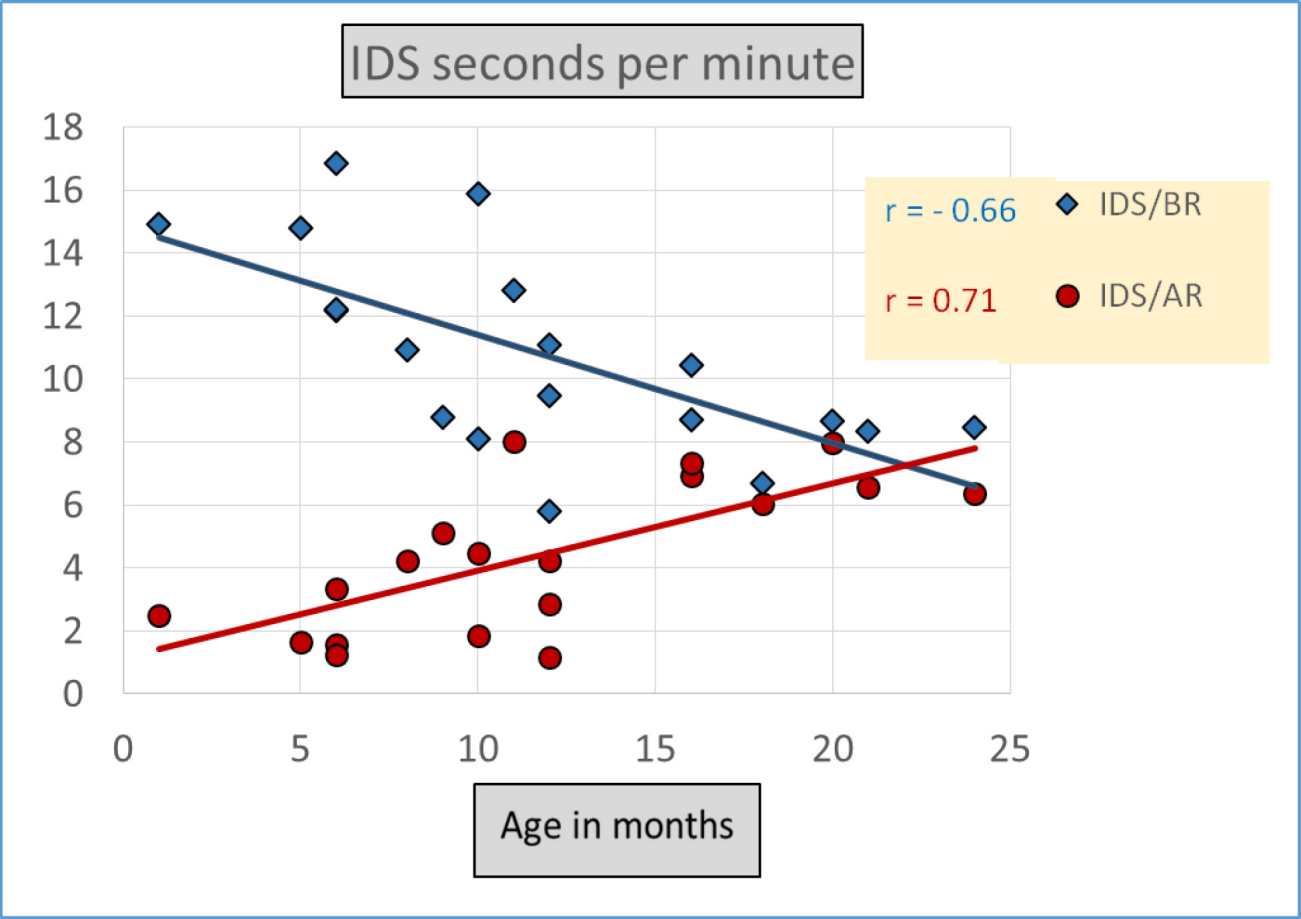
Average duration in *Seconds per Minute* of maternal IDS/BR and IDS/AR by Age. Both groups showed more *IDS/BR* in Seconds per Minute than *IDS/AR*. Results also showed *less IDS/BR* for older infants and *more IDS/AR* in Seconds per Minute for older infants.

Another way to measure *amount* of IDS, in addition to Utterances per Minute during recordings, is by Seconds per Minute occupied by IDS during recordings. Comparing analyses for these two measures, we take account of possible differences in the durations of IDS utterances that might yield different patterns of results in the two cases. Table 2 records results on Seconds per Minute, illustrating the similar but not identical outcomes to those of the Utterances-per-Minute analysis. The American mothers tended to produce *fewer* Seconds per Minute of IDS with older infants, whereas Lebanese mothers produced *more* Seconds per Minute of IDS with older infants. Fig 3 suggests that both language groups produced more IDS/BR Seconds per Minute at the *younger* ages, and more IDS/AR at the older ages. Using the same MANCOVA design as for the Utterances-per-Minute analysis, the Seconds-per-Minute analysis remained statistically significant for Register, Wilks’ Lambda = .43, *F* (1,34) = 47.40, *p* < .001, partial eta squared = .58, and Register by Age, Wilks’ Lambda = .68, *F* (2,33) = 16.62, *p* < .001, partial eta squared = .32, again indicating that IDS/BR was utilized more than IDS/AR and that the extent of IDS/BR usage was higher with infants at younger ages. No significant interaction of Register by Language was found, Wilks’ Lambda = .99, *F* (3, 32) = .12, *p* = .74, partial eta squared = .00. The Language effect, however, did not reach statistical significance, *F* (3, 32) = 2.0, *p* = .17, partial eta squared = .05 in the Seconds-per-Minute analysis, though it corresponded, as in the case of the Utterances-per-Minute analysis, to a larger amount of IDS by the Lebanese than the American mothers (8% more IDS/BR and 40% more IDS/AR).

The differences between these two analyses would not occur if average utterance durations had not varied across groups. Table 2 illustrates that Duration in *Seconds per Utterance* varied in the two Language groups and across the Registers. The MANCOVA results for the Seconds-per-Utterance analysis were statistically significant for Register, Wilks’ Lambda = .85, *F* (1,34) = 6.38, *p* < .05, partial eta squared = .15, indicating that IDS/BR was utilized more than IDS/AR. However, the Register by Age interaction did not reach statistical significance, Wilks’ Lambda = 1.0, *F* (2,33) = .01, *p* = .92, partial eta squared = .00, suggesting that the extent of IDS/BR usage did not differ between infants at younger and older ages. Likewise, no significant interaction of Register by Language was found, Wilks’ Lambda = 1.0, *F* (3, 32) = .01, *p* = .94, partial eta squared = .00. The Language effect did not reach statistical significance, *F* (3, 32) = 2.09, *p* = .16, partial eta squared = .06, though it corresponded to longer utterances by the American than the Lebanese mothers (9% longer IDS/BR and 12% longer IDS/AR). Obviously, since Lebanese mothers’ voices in IDS occupied more time in the recordings than American mothers’ voices, the greater number of Utterances per Minute of the Lebanese mothers more than counterbalanced the tendency for American mothers to use longer utterances. The tendency for American mothers to use longer utterances was not, however, consistent across Age.

## Discussion

While others have reported differences in patterns of IDS for different developmental levels [52] and across cultural contexts [8], this exploratory study is the first to report quantitatively on the role of language differences in rate of IDS and on use of different registers *within* IDS (IDS/BR vs IDS/AR) for different languages. The results revealed the expected higher frequency of IDS/BR compared to IDS/AR for both languages, but the high rate of IDS/AR was unexpected, with mothers producing 33% of their utterances in IDS/AR. Especially surprising was the fact that mothers at all infant ages and in both languages produced at least some IDS/AR in these 10-minute samples. In contrast, the higher rate of IDS/AR at older infant ages for both language groups was not surprising, presumably because infants become increasingly able to understand adult speech as they get older [53] and thus may not need the facilitative support in comprehension brought about by features inherent in IDS/BR [37, 38].

The key difference in IDS between the two language groups was the higher rate in Utterances per Minute of IDS of the Lebanese as opposed to the American mothers. Here, we can only speculate about possible reasons. [54] suggested that cultures vary in the ways parents view infants as communication partners. Considerable writing in the realm of cross-cultural parent-infant interaction supports this view (see review in [55, 56]), for example contending that cultural differences are rooted in culturally-specific views on how to communicate with infants. Americans are viewed in this context as particularly supportive of personal independence and assertiveness and thus are thought to favor interactions with infants and children that seek to foster such independence from as early as possible. Many non-Western cultures, in contrast, are viewed as more supportive of *inter*dependence, courtesy, and social interconnectedness. In keeping with this reasoning, we speculate that the Lebanese mothers may have talked more to their babies (i.e., used more IDS) simply as a reflection of a somewhat greater inclination to foster *inter*dependence in the infants.

Other possible reasons for more IDS Utterances per Minute in the Lebanese mothers (differences between the languages in learnability, differences in cultural attitudes about physical as opposed to verbal interaction, etc.) are similarly speculative. Our inclination is to leave such issues to future research. The current exploratory study was underpowered (with only 19 infants per group across a two-year age span and a single recording) to yield very strong conclusions and does not provide a basis to evaluate many of the possible explanations that might be entertained.

Further, our thoughts about language differences in IDS are complicated by the fact that the differences in amount of IDS for the two language groups were statistically significant only for the Utterances-per-Minute analysis. The Seconds-per-Minute analysis also showed more IDS from the Lebanese mothers, but the Language difference in this case was not statistically significant—the pattern was affected by a tendency of American mothers to produce slightly longer utterances and by the fact that this difference across the languages varied with age. Both analyses showed a strong Register effect (more IDS/BR than IDS/AR), and both showed a strong interaction of Register with Age, a pattern that hints that by the end of the second year, IDS/AR could be taking over as the predominant form of IDS in both language groups.

## Limitations

This study was opportunistic, resulting in samples that were less than perfectly matched. It also relied on cross-sectional data, relatively brief durations of mother-infant interactions, and slightly different instrumentation for recording mother-infant interactions across the Lebanese and American cultures. The location of the recordings (home versus laboratory) also differed across the two groups. The impact of this difference is less likely to change the pattern of results we obtained, however, as research suggests similar results in mother-child interactions across the home and laboratory settings [57, 58], especially when mothers from different groups are instructed to interact with their infants [59]. In this study, both Lebanese and American mothers were instructed to interact with their infants. Of similar importance is the limited number of infants studied. A fully longitudinal effort would also be advisable. Improvement on such factors would undoubtedly improve generalizability of the findings.

Further, we only focused on maternal input and did not address the infant’s contribution to the interaction. This is not trivial, especially considering research on mother-child interaction and the bidirectional role that each member of the dyad plays in the process of early vocal development [60, 61]. Importantly, such efforts have the potential for situating vocal development and interaction styles as clinical markers, and hopefully they could in the future help guide early detection of developmental anomalies such as autism spectrum disorders (ASD) and the mechanisms contributing to this developmental derailment in the first year of life. Future studies should include not only the mother-infant dyad, but also other interactors from various age, gender, or class groups that might play a significant role in the input to which the infant is exposed. We would hope in our own future efforts to also take account of the role of overheard speech, which is language spoken not to infants, but among caregivers who are in earshot of the infant.

A final thought is that it would be enormously preferable to obtain recordings in both audio and video so that coding could be conducted across a variety of modalities of parent and infant action, encompassing vocalization, gaze, facial affect, gesture, posture, and physical proximity. A more comprehensive approach including these sorts of improvements would allow a much fuller portrayal, illuminating the role of IDS/BR and IDS/AR in supplying human infants with a human-specific niche for language learning, and presumably with culturally-specific forms of the human language environment.
